# Association between postoperative acute kidney injury and mortality after plastic and reconstructive surgery

**DOI:** 10.1038/s41598-022-24564-0

**Published:** 2022-11-21

**Authors:** Ha Min Sung, Ah Ran Oh, Minsu Jung, Hara Kim, Sooyeon Lee, Dahye Cha, Jungchan Park

**Affiliations:** 1 Link Plastic Surgery Clinic, Seoul, 06120 Korea; 2 Department of Anesthesiology and Pain Medicine, Samsung Medical Center, Sungkyunkwan University School of Medicine, 81 Irwon-Ro, Gangnam-Gu, Seoul, 06351 Korea; 3 Department of Anesthesiology and Pain Medicine, Kangwon National University Hospital, Chuncheon, 24341 Korea; 4 Department of Biomedical Sciences, Ajou University Graduate School of Medicine, Suwon, 16499 Korea

**Keywords:** Health care, Nephrology, Risk factors

## Abstract

Acute kidney injury (AKI) is a common postoperative disorder that is associated with considerable morbidity and mortality. Although the role of AKI as an independent risk factor for mortality has been well characterized in major surgeries, its effect on postoperative outcomes in plastic and reconstructive surgery has not been evaluated. This study explored the association between postoperative AKI and mortality in patients undergoing plastic and reconstructive surgery. Consecutive adult patients who underwent plastic and reconstructive surgery without end-stage renal disease (*n* = 7059) at our institution from January 2011 to July 2019 were identified. The patients were divided into two groups according to occurrence of postoperative AKI: 7000 patients (99.2%) in the no AKI group and 59 patients (0.8%) in the AKI group. The primary outcome was mortality during the first year, and overall mortality and 30-days mortality were also compared. After inverse probability weighting, mortality during the first year after plastic and reconstructive surgery was significantly increased in the AKI group (1.9% vs. 18.6%; hazard ratio, 6.69; 95% confidence interval, 2.65–16.85; *p* < 0.001). In this study, overall and 30-day mortalities were shown to be higher in the AKI group, and further studies are needed on postoperative AKI in plastic and reconstructive surgery.

## Introduction

Advances in surgical techniques and anesthetic management have significantly increased the safety of plastic and reconstructive surgery^1^. As a result, mortality in this area is infrequent but remains a costly outcome. As the number and complexity of surgeries in this field continue to increase, it becomes important to investigate risk factors to minimize the incidence of mortality.

Acute kidney injury (AKI) occurs in approximately 1 to 5% of all hospitalized patients and is increasingly prevalent^2,3^. It is a common postoperative complication and strongly associated with prolonged hospital stay and increased morbidity and mortality^4,5^. Especially, the occurrence of AKI in the week after surgery appears to be of prognostic significance beyond the acute surgical episode, related to the development of chronic kidney disease and late death^6^. Despite the importance of AKI as a complication after surgery, previous studies have focused on AKI in major surgical procedures such as cardiac or major non-cardiac surgery^7–9^. In addition, postoperative mortality was shown to increase with severity of AKI^10^, and monitoring for AKI tends to be neglected in low-risk procedures, despite previous report on increased complication even by small acute increase of creatinine concentration after non-cardiac surgery^9,11,12^. Plastic and reconstructive surgery is a less invasive procedure where the effect of AKI on postoperative outcomes has scarcely been reported. In this study, we aimed to report the incidence of postoperative AKI in patients undergoing plastic and reconstructive surgery as well as the recovery from AKI before discharge. We also evaluated whether postoperative AKI is associated with mortality despite expected low incidence after plastic and reconstructive surgery. Our findings may be helpful for plastic surgeons to reduce postoperative mortality and deliver surgical techniques more safely to patients.

## Materials and methods

This study was a retrospective cohort study approved by the Institutional Review Board at Samsung Medical Center (SMC 2020–09-001). The requirement for written informed consent was waived by the Institutional Review Board at Samsung Medical Center, because the data were collected retrospectively in de-identified form. We conducted this research following the Declaration of Helsinki and reported results according to the Strengthening the Reporting of Observational Studies in Epidemiology guideline.

### Study population and data collection

We enrolled consecutive adult patients undergoing plastic and reconstructive surgery at Samsung Medical Center from January 2011 to July 2019. After excluding patient with end-stage renal disease, patients were divided into two groups according to occurrence of postoperative AKI. We curated the medical records of study patients in a de-identified form using our electronic archive system “Clinical Data Warehouse Darwin-C.” This electronic system allowing researchers to review and retrieve data for investigation from electronic hospital records of more than 2.2 million surgeries, one billion laboratory findings, 100 million disease codes, and 200 million prescriptions. The results of blood laboratory tests were automatically curated. In this system, mortality data from other than our institution are consistently validated and updated according to the National Population Registry of the Korea National Statistical Office, so there was no loss of follow-up for mortality. Medical records were manually reviewed by independent investigators who were blinded to the development of postoperative AKI.

### Study outcomes and definitions

The primary endpoint of this study was one-year mortality after surgery. Secondary outcomes were mortality during overall follow-up period and 30-day mortality. The occurrence of AKI was defined as either an increase in serum creatinine ≥ 0.3 mg/dL within 48 h after surgery or an increase to ≥ 1.5 times baseline within 7 days based on the Kidney Disease: Improving Global Outcomes criteria^13^. For the baseline, we used creatinine level obtained during preoperative evaluation within 3 months before surgery. According to the institutional protocol, patients with elevated creatinine level were rechecked at admission for operation. The Charlson comorbidity index was calculated using the 10th revision of the International Statistical Classification of Diseases and Related Health Problems (ICD-10)^14^.

### Statistical analysis

Baseline characteristics are presented as mean (± standard deviation) or median (interquartile range) for continuous variables and numbers with percentages for categorical variables. Differences between two groups were compared using the t-test or the Mann–Whitney test for continuous variables and χ2 or Fisher’s exact test for categorical variables. The following covariates with the absolute standardized mean difference (ASD) > 10% were selected for multivariable analysis: age, sex, smoking, hypertension, Charlson comorbidity index (CCI), preoperative creatinine and hemoglobin level, operative duration, general anesthesia, and type of surgery. To reduce further selection bias and unavoidable confounding factors, we conducted rigorous adjustment for differences in baseline patient characteristics using inverse probability weighting (IPW). In this technique, the inverse probability weights were defined as the reciprocal of propensity scores, and the ASD under 10% was deemed as an achievement of balance between the groups^15^. Variables with ASD over 10% after IPW technique were further adjusted with regression method. Differences in mortality were presented as hazard ratio (HR) and 95% confidence interval (Cl) using conventional Cox and weighted Cox proportional hazards analysis with IPW. Kaplan–Meier estimates were used to construct survival curves and were compared using the log-rank test. We also performed subgroup analysis according to types of surgery, general anesthesia, renal disease, preoperative elevation. The results are presented as forest plots. All statistical analyses were performed with R 4.0.2 (Vienna, Austria; http://www.R-project.org/). All tests were two-tailed, and *p* < 0.05 was considered statistically significant.

### Ethics approval and consent to participate

This retrospective cohort study was approved by the Institutional Review Board at Samsung Medical Center (SMC 2020-09-001). The requirement for written informed consent was waived because the data were collected retrospectively in de-identified form.

## Results

### Baseline characteristics

From January 2011 to July 2019, a total of 7089 patients underwent plastic and reconstructive surgery and all patients had an available preoperative creatinine values. After excluding patient with end-stage renal disease, 7,059 patients were enrolled for analysis.

The median duration of hospital stay was 8 days (interquartile range 6–13). We divided the study patients into two groups according to occurrence of postoperative AKI: 7000 patients (99.2%) in the no AKI group and 59 patients (0.8%) in the AKI group. The most of AKI group was recovered from AKI except for 5 patients with persistent creatinine elevation from baseline. The patients’ baseline characteristics are summarized in Table 1. The AKI group tended to show higher values in most demographic variables and underlying disease but had shorter operative time and lower incidence of general anesthesia.

**Table 1:** Baseline characteristics according to postoperative acute kidney injury (AKI).

	No AKI (N = 7000)	AKI (N = 59)	*p*-value	ASD	IPW ASD
*Age, years	45.0 (36–55)	62.0 (53.5–69.5)	< 0.001	98.5	35.2
*Male	2105 (30.1)	41 (69.5)	< 0.001	85.8	25.2
*Smoking	720 (10.3)	12 (20.3)	0.02	28.2	0.6
*Alcohol	1753 (25.0)	16 (27.1)	0.83	4.7	3.1
*Hypertension	389 (5.6)	12 (20.3)	< 0.001	45.1	14.3
*Charlson comorbidity index	0.3 (± 1.01)	1.93 (± 2.74)	< 0.001	77.0	35.3
Myocardial infarction	9 (0.1)	3 (5.1)	< 0.001	31.5	
Heart failure	20 (0.3)	2 (3.4)	0.002	23.3	
Peripheral vascular disease	18 (0.3)	0	> 0.99	7.4	
Cerebrovascular disease	103 (1.5)	7 (11.9)	< 0.001	4.6	
Dementia	1 (0.0)	0	> 0.99	1.7	
Chronic pulmonary disease	4 (0.1)	0	> 0.99	3.4	
Rheumatic disease	60 (0.9)	1 (1.7)	> 0.99	5.4	
Peptic ulcer disease	0	0	> 0.99	< 0.1	
Mild liver disease	231 (3.3)	7 (11.9)	0.001	32.8	
Diabetes without complication	247 (3.5)	15 (25.4)	< 0.001	65.5	
Diabetes with complication	89 (1.3)	11 (18.6)	< 0.001	60.6	
Hemiplegia	19 (0.3)	2 (3.4)	0.001	23.4	
Renal disease	84 (1.2)	16 (27.1)	< 0.001	80.1	
Any malignancy	353 (5.0)	0	0.14	32.6	
Moderate to severe liver disease	4 (0.1)	1 (1.7)	0.02	17.6	
Metastatic solid tumor	0	0	> 0.99	< 0.1	
Human immunodeficiency virus	1 (0.0)	0	> 0.99	1.8	
**Preoperative blood test**					
*Hemoglobin, g/dL	13.1 (± 1.8)	12.7 (± 1.7)	0.05	26.6	21.3
*Creatinine, mg/dL	0.78 (± 0.45)	2.39 (± 2.49)	< 0.001	89.9	27.9
**Operative variables**					
*Duration, hours	3.3 (1.5–4.8)	1.2 (0.5–3.6)	< 0.001	12.8	10.2
*General anesthesia	6549 (93.6)	37 (62.7)	< 0.001	80.4	2.7
*Emergency surgery	442 (6.3)	9 (15.3)	0.01	29.1	8.3
*Type			0.7	18.3	39.6
Head and Neck	1009 (14.4)	12 (20.3)			
Trunk and extremities	1328 (19.0)	11 (18.6)			
Breast	3177 (45.4)	25 (42.4)			
Aesthetic surgery	167 (2.4)	2 (3.4)			
Mass excision	1319 (18.8)	9 (15.3)			

Values are n (%), mean (± standardized difference), or median (interquartile range).

*ASD* absolute standardized mean difference, *IPW* inverse probability weighting.

*Following variables were retained for IPW adjustment.

**Figure 1:**
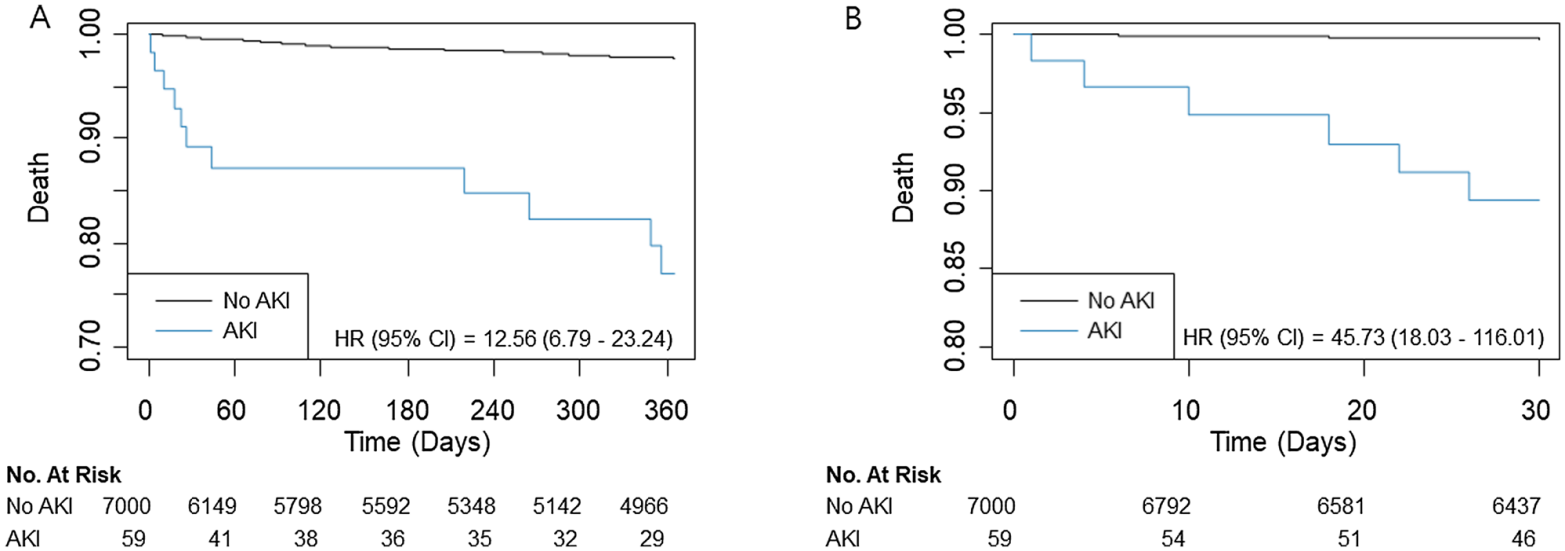
Kaplan–Meier curves of all-cause mortality during (**A**) one-year and (**B**) 30-day follow-ups.

**Figure 2:**
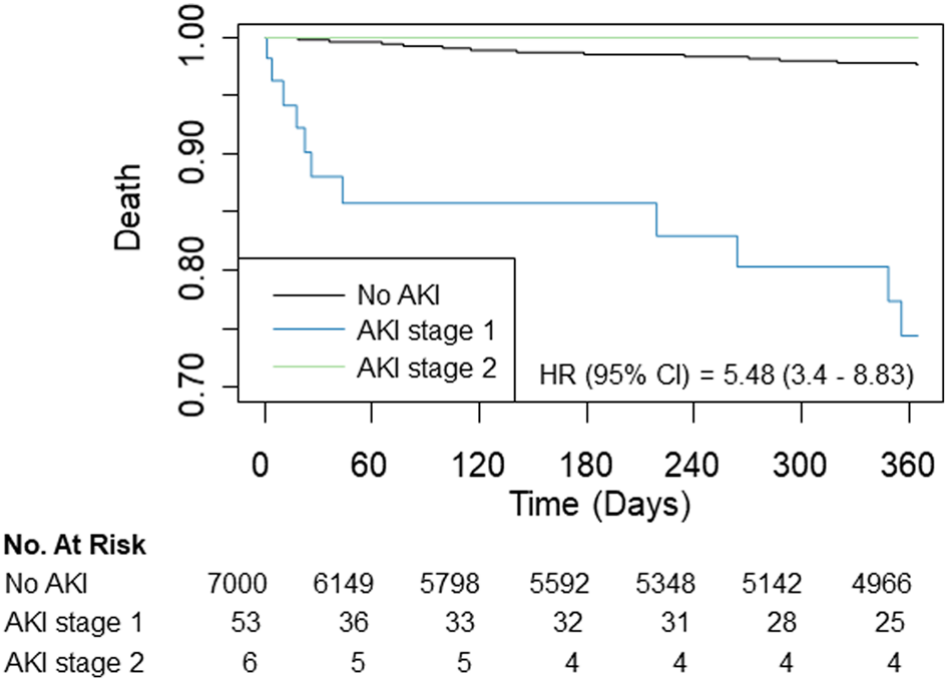
Kaplan–Meier curves of all-cause mortality during one-year follow-up according to stages of AKI.

**Figure 3:**
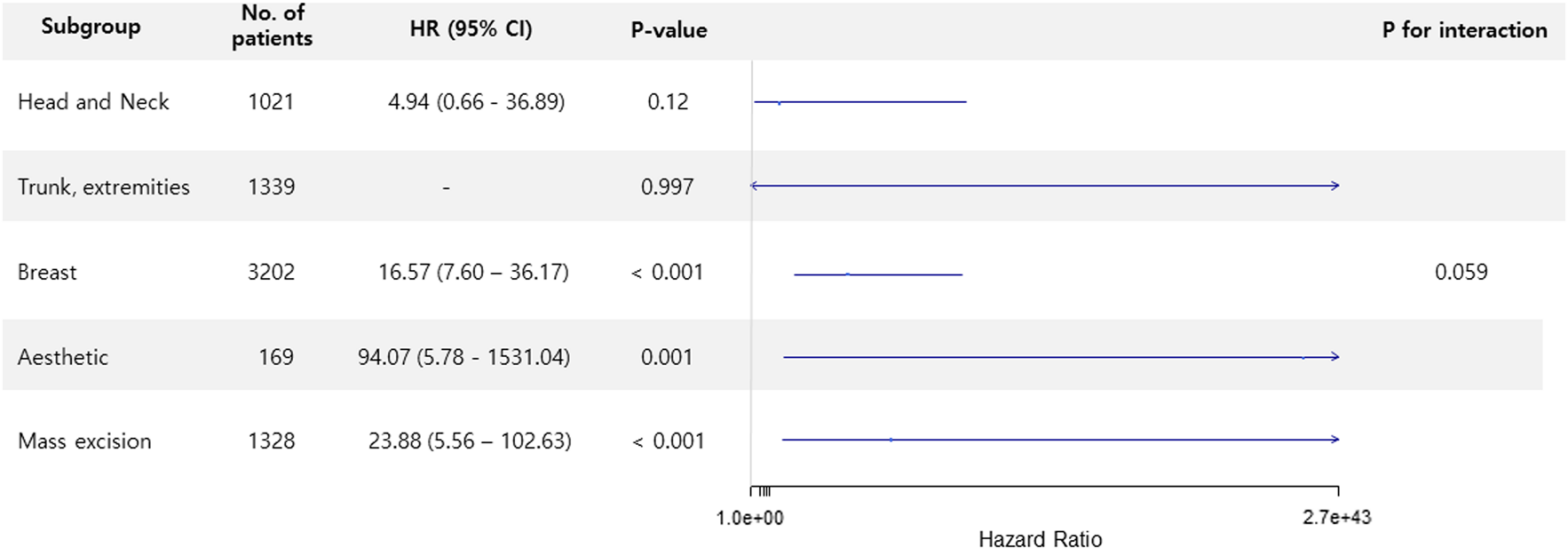
Forest plot of subgroup analysis according to type of surgery.

**Figure 4:**
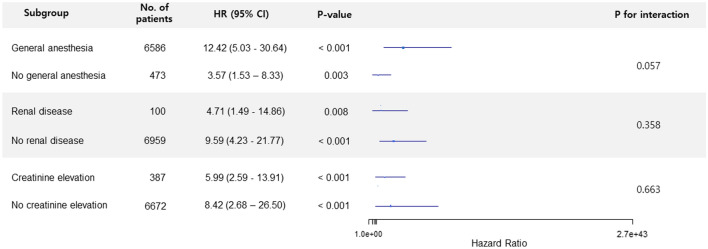
Forest plot of subgroup analysis according to general anesthesia and previous renal disease.

The mortality rate was 2.0% (142/7,059) during the first year after surgery, 4.8% (341/7059) during the overall follow-up period, and 0.33% (23/7059) for 30 days. The median follow-up duration for one-year mortality was 365 days (interquartile range: 261–365 days). In multivariable analysis, mortality during the first year was significantly higher in AKI group (1.9% vs. 18.6%; HR, 3.34; 95% CI 1.69–6.59; *p* = 0.001) (Table 2). Similar results were found in mortality during the overall follow-up period (4.6% vs. 28.8%; HR, 3.39; 95% CI 1.99–5.77; *p* < 0.001) and 30-day mortality (0.2% vs. 10.2%; HR, 9.08; 95% CI 2.94–28.02; p < 0.001). This trend persisted after IPW adjustment (HR 6.69, 95% CI 2.65–16.85, *p* < 0.001 for one-year mortality; HR 4.94, 95% CI 2.16–11.27, *p* < 0.001 for overall mortality and HR 21.40, 95% CI 5.41–84.66, *p* < 0.001 for 30-day mortality). Survival curves are presented in Figs. 1 and 2. The subgroup analysis was conducted according to types of surgery, general anesthesia, renal disease, preoperative creatinine elevation (Figs. 3 and 4), and there was no significant interaction observed.

**Table 2:** Mortalities according to postoperative acute kidney injury (AKI).

	No AKI (N = 7000)	AKI (N = 59)	Unadjusted HR (95% CI)	*p-*value	Adjusted HR (95% CI)	*p-*value	IPW Adjusted HR (95% CI)	*p-*value
One-year mortality	131 (1.9)	11 (18.6)	12.56 (6.79–23.24)	< 0.001	3.34 (1.69–6.59)	0.001	6.69 (2.65–16.85)	< 0.001
Overall mortality	324 (4.6)	17 (28.8)	9.61 (5.90–16.66)	< 0.001	3.39 (1.99–5.77)	< 0.001	4.94 (2.16–11.27)	< 0.001
30-days mortality	17 (0.2)	6 (10.2)	45.73 (18.03–116)	< 0.001	9.08 (2.94–28.02)	< 0.001	21.40 (5.41–84.66)	< 0.001

*IPW* inverse probability weighting, *HR* hazrd ratio, *CI* confidence interval.

Multivariable analysis retained age, male, hypertension, Charlson comorbidity index, preoperative creatinine and hemoglobin levels, operative duration, general anesthesia, emergency operation, and type of surgery.

IPW adjustment analysis retained age, male, hypertension, smoking, alcohol, Charlson comorbidity index, preoperative creatinine and hemoglobin levels, operative duration, general anesthesia, emergency operation, and type of surgery, and variables with absolute standard mean difference over 10% were further adjusted with regression method.

For additional analysis, we stratified the AKI group according to stage. Among 59 patients in AKI group, 53 (89.8%) were stage 1 and 6 (10.2%) were stage 2. The baseline characteristics of AKI group according to stage are presented in Table 3.

**Table 3:** Baseline characteristics and mortalities according to stages of postoperative acute kidney injury (AKI).

	AKI stage 1 (N = 53)	AKI stage 2 (N = 6)	*p*-value
Age, years	62.0 (54.0–70.0)	56.0 (47.0–62.0)	0.19
Male	38 (71.7)	3 (50.0)	0.53
Smoking	11 (20.8)	1 (16.7)	0.11
Alcohol	13 (24.5)	3 (50.0)	0.55
Hypertension	10 (18.9)	2 (33.3)	0.33
Charlson comorbidity index	1.93 (± 2.67)	2.00 (± 3.52)	0.95
Myocardial infarction	3 (5.7)	0	N/A
Heart failure	2 (3.8)	0	N/A
Peripheral vascular disease	0	0	N/A
Cerebrovascular disease	6 (11.3)	1 (16.7)	> 0.99
Dementia	0	0	N/A
Chronic pulmonary disease	0	0	N/A
Rheumatic disease	1 (1.9)	0	N/A
Peptic ulcer disease	0	0	N/A
Mild liver disease	6 (11.3)	1 (16.7)	> 0.99
Diabetes without complication	13 (24.5)	2 (33.3)	> 0.99
Diabetes with complication	11 (20.8)	0	N/A
Hemiplegia	2 (3.8)	0	N/A
Renal disease	16 (30.2)	0	N/A
Any malignancy	0	0	N/A
Moderate to severe liver disease	0	1 (16.7)	0.18
Metastatic solid tumor	0	0	N/A
Human immunodeficiency virus	0	0	N/A
**Preoperative blood test**			
Hemoglobin, g/dL	12.6 (± 1.7)	13.5 (± 1.3)	0.2
Creatinine, mg/dL	2.58 (± 2.56)	0.68 (± 0.15)	0.11
**Operative variables**			
Duration, hours	1.2 (0.5–3.9)	1.3 (0.6–2.3)	0.95
General anesthesia	32 (60.4)	5 (83.3)	0.51
Emergency surgery	9 (17.0)	0	N/A
**Type**			
Head and neck	12 (22.6)	0	
Trunk and extremities	10 (18.9)	1 (16.7)	
Breast	21 (39.6)	4 (66.7)	
Aesthetic surgery	2 (3.8)	0	
Mass excision	8 (15.1)	1 (16.7)	
**Mortalities**			
One-year mortality	11 (20.8)	0	0.49
Overall mortality	16 (30.2)	1 (16.7)	0.83
30-day mortality	6 (11.3)	0	0.88

Values are n (%), mean (± standardized difference), or median (interquartile range).

## Discussion

The main finding of this study was that postoperative AKI was associated with an enormous increase of one-year, overall, and 30-days mortality after plastic and reconstructive surgery. The incidence of AKI was 0.9%, of which 88% was stage 1. In this study, AKI was shown as a risk factor for mortality after plastic and reconstructive surgery, but further studies are required for AKI to be used in the prediction of short-term and long-term mortality after plastic and reconstructive surgery owing to following issues.

While a myriad of studies has discussed the relationship between postoperative AKI and mortality, there has been little attention on less invasive procedures such as plastic and reconstructive surgery. Our analysis showed that the incidence of postoperative AKI was approximately 0.9% after plastic and reconstructive surgery, which was much lower than values reported in other types of surgery^16–18^. This might be related to the generally healthier status of patients undergoing plastic and reconstructive surgery with fewer underlying diseases and medications compared to patients undergoing other surgeries. Our study patients had low mean CCI value < 2 in both groups.

In our study, the vast majority of our AKI group were stage 1 (88%). It is well known that the risk of mortality increases with increasing severity of AKI^19,20^. According to a previous study, patients with KDIGO stage 1 AKI did not demonstrate substantially higher mortality than their non-AKI controls^20^, suggesting that mild AKI does not expose patients to worse outcome. However, our result showed that the risk for death was considerably increased compared to that of patients without AKI. This result can be explained by the presumption that AKI mainly occurred in vulnerable patients with many underlying diseases, implying that even small changes in kidney function in these patients caused high mortality^2^. Furthermore, the occurrence of AKI might have led to rapid exacerbation, which contributed more to short-term mortality than to long-term mortality. However, future studies addressing the prognostic value of postoperative AKI by stage are required because statistical power might have been reduced due to the low incidence of stage 2 or higher in our analysis.

Another issue to consider is the effect of preoperative renal condition. The AKI group showed higher incidence of previous renal disease as well as higher level of preoperative creatinine. Preoperative creatinine was not fully balanced after an initial adjustment with IPW which required further adjustment using regression method. Additionally, we conducted a subgroup analysis for previous renal disease and preoperative creatinine elevation. Although we could not find any significant interaction, it is still difficult to conclude whether the mortality is associated with postoperative AKI per se or baseline kidney condition. So, further prospective study is needed.

In subgroup analysis, the association between postoperative AKI and one-year morality was not significantly interacted with other variables. However, the increase of risk was higher in patients undergoing general anesthesia. This finding suggests that the risk of AKI can be influenced by anesthesia-induced hemodynamic changes such as venodilation or reduced arterial tone. A study on healthy volunteers found that sympathetic block by epidural anesthesia did not change renal blood flow significantly^21^. In addition, a meta-analysis found that the incidence of AKI was lower for neuraxial anesthesia compared with general anesthesia^22^. These results might be related to the greater chance of hypotension and preload reduction in general anesthesia, which compromise renal perfusion and contribute to the occurrence of AKI.

Limitations should be recognized in interpreting the results of this analysis. First, this was a single-center retrospective study, so we could not exclude confounding caused by non-measured factors regardless of rather rigorous statistical adjustment. Preoperative kidney disease could not be specified according to stage, and the effect of preoperative kidney condition might have affected the result. Also, due to the long study period, the development of both surgical techniques and postoperative management could have biased the results. Second, our patients had only low stage AKI, so this study could not assess the impact of AKI according to stage. Moreover, the definition of AKI has changed and evolved over the years, complicating assessment of incidence and prevalence. In addition, there are three classification systems of AKI that have different diagnostic criteria. Therefore, our results cannot be generalized when applying different classification criteria in different clinical settings. Despite these limitations, this is the first study to report an association between postoperative AKI and mortality in plastic and reconstructive surgery.

In conclusion, this study demonstrated that postoperative AKI can be associated with increased risk of mortality after plastic and reconstructive surgery, but further studies are needed for clinical application of our findings.

## Data Availability

All relevant data are within the paper.

## Abbreviation

AKI: Acute kidney injury
